# The Overlap between Irritable Bowel Syndrome and Non-Celiac Gluten Sensitivity: A Clinical Dilemma

**DOI:** 10.3390/nu7125541

**Published:** 2015-12-10

**Authors:** Archita Makharia, Carlo Catassi, Govind K. Makharia

**Affiliations:** 1 Department of Gastroenterology and Human Nutrition, All India Institute of Medical Sciences, Ansari Nagar, New Delhi 110029, India; govindmakharia@gmail.com; 2 Department of Pediatrics, Università Politecnica delle Marche, 60123 Ancona, Italy; c.catassi@univpm.it

**Keywords:** celiac disease, wheat allergy, intestine, pathogenesis, gluten-related disorders

## Abstract

The spectrum of gluten-related disorders has widened in recent times and includes celiac disease, non-celiac gluten sensitivity, and wheat allergy. The complex of symptoms associated with these diseases, such as diarrhea, constipation or abdominal pain may overlap for the gluten related diseases, and furthermore they can be similar to those caused by various other intestinal diseases, such as irritable bowel syndrome (IBS). The mechanisms underlying symptom generation are diverse for all these diseases. Some patients with celiac disease may remain asymptomatic or have only mild gastrointestinal symptoms and thus may qualify for the diagnosis of IBS in the general clinical practice. Similarly, the overlap of symptoms between IBS and non-celiac gluten sensitivity (NCGS) often creates a dilemma for clinicians. While the treatment of NCGS is exclusion of gluten from the diet, some, but not all, of the patients with IBS also improve on a gluten-free diet. Both IBS and NCGS are common in the general population and both can coexist with each other independently without necessarily sharing a common pathophysiological basis. Although the pathogenesis of NCGS is not well understood, it is likely to be heterogeneous with possible contributing factors such as low-grade intestinal inflammation, increased intestinal barrier function and changes in the intestinal microbiota. Innate immunity may also play a pivotal role. One possible inducer of innate immune response has recently been reported to be amylase-trypsin inhibitor, a protein present in wheat endosperm and the source of flour, along with the gluten proteins.

## 1. Introduction

Irritable bowel syndrome (IBS) is a chronic functional disorder of the gastrointestinal tract and it is characterized by the presence of abdominal pain or discomfort in association with a change in bowel habit and with a sensation of bloating [1,2,3]. IBS is a common disease in the general population and its prevalence varies from country to country and the criteria used for the diagnosis of IBS [1,2,3,4,5]. A meta-analysis including 81 studies and 260,960 subjects, showed that the pooled prevalence of IBS was 11.2% (95% confidence interval, 9.8%–12.8%) [4]. The prevalence of IBS in Asian countries varies from 6.5% to 11.1% [5]. Two population-based studies from India have estimated that approximately 4% of Indians have IBS [6,7]. There are certain features that help clinicians make a diagnosis of IBS, such as long-standing symptoms, alterations in bowel habit, abdominal pain, absence of constitutional symptoms, and absence of alarm features such as weight loss, anorexia, gastrointestinal bleeding and fever. None of these features are, however, very specific for IBS and similar symptoms may occur in many other gastrointestinal diseases. The diagnosis of IBS is based on clinical characteristics and most guidelines do not recommend extensive investigations for the diagnosis of IBS, except in the presence of alarming symptoms [1,2,3]. Gastrointestinal diseases such as giardiasis, lactose intolerance, small intestinal bacterial overgrowth and celiac disease produce symptoms mimicking those of IBS and they may easily be misdiagnosed as IBS [1,2,3,8,9,10,11]. Gluten-related disorders have recently been recognized as commonly mimicking IBS [12,13,14,15,16,17,18,19]. In the following section, we review the overlap and inter-relationship between IBS and gluten-related disorders.

## 2. Spectrum of Gluten-Related Disorders

The spectrum of gluten-related disorders has widened in recent times and includes celiac disease (CeD), non-celiac gluten sensitivity (NCGS), and wheat allergy [20,21,22,23,24]. While CeD affects one percent of the general population, the epidemiology of NCGS is still evolving [25,26,27]. Some studies suggest that the prevalence of NCGS in the general population may be up to 5% although other studies suggest lower prevalence [18,28,29,30]. The reason why some patients develop CeD and others NCGS is not well known. While up to 90% of patients with CeD have specific genetic susceptibility represented by genes of the major histocompatibility complex (HLA-DQ2 and -DQ8 haplotype, HLA: Human leukocyte antigen), these genes show up in NCGS patients at a much lower level of 50%, but a level of 30% is characteristic of the general population [14,18,29,30]. Furthermore while both adaptive and innate immune responses are involved in CeD, in NCGS, innate immune response to gluten peptides may be the predominant immune reaction [17,18,22,31,32]. Furthermore, it is possible that the gut microbiota play a role in driving the immune process in certain individuals towards CeD (if they have specific genetic susceptibility factors) while driving other individuals to NCGS. By producing gluten-degrading enzymes, oral and intestinal microbiota might convert an immunogenic gluten-peptide to non-immunogenic peptide thus leading to a cascade of events directed towards NCGS [17,18,22,29,30,31,32].

## 3. Does CeD Exist in Patients with IBS?

The spectrum of manifestations of CeD is wide and ranges between classic CeD having typical signs and/or symptoms of malabsorption (such as diarrhea, weight loss, vitamin deficiencies, or malnutrition) and non-classic CeD having non-GI symptoms such as short stature, anemia, infertility, or osteopenia/osteoporosis [20,21,24]. Furthermore, some patients with CeD remain asymptomatic or have only mild GI symptoms. While in some patients the phenotype of the disease is fully expressed, in many others the disease is expressed only in the milder form [20,33,34,35,36]. Many such patients with CeD having mild gastrointestinal symptoms may fulfill the criteria for the diagnosis of IBS and they may be diagnosed and treated as IBS in the general clinical practice [20,21,24,33,34,35,36].

Overall 4% of patients meeting criteria for IBS are confirmed to have CeD based on a meta-analysis including 14 studies [37]. Specifically, odds of having CeD in patients meeting diagnostic criteria for IBS is 4.34 (95% confidence interval, 1.78–10.6) in comparison to controls [37]. Additionally almost one third of patients with CeD who already are on treatment with a gluten-free diet continue to have IBS-type symptoms. Furthermore, evidence now suggests that testing for CeD in patients with diarrhea-predominant IBS is cost-effective if the prevalence of CeD in the general population is above 1% [38]. Based on the evidences of overlap in the symptomatology of IBS and CeD, the American College of Gastroenterology Task Force has recommended routine serologic screening for CeD in patients with diarrhea-predominant IBS or IBS with a mixed bowel pattern [39].

While the abovementioned meta-analysis showed a higher prevalence of CeD in patients with IBS, it is important to note that data from the studies from US and India have not confirmed this. In a prospective study from United States, including 492 patients with IBS symptoms, the prevalence of CeD was 0.4% [14]. In a study from India including 362 patients with IBS, we observed that only 3 (0.8%) patients had biopsy confirmed CeD, which was comparable to the prevalence of CeD in the general population [40]. Interestingly, IgA anti-tissue transglutaminase antibody was positive in 19 other patients with IBS, they however had evidence of either no enteropathy or mild enteropathy (Modified Marsh grade 0 or 1) and hence qualifying for a diagnosis of potential CeD. Additionally, a larger number (28.7%) of them had a positive anti-gliadin antibody test suggesting presence of some form of gluten sensitivity in them [40].

In summary, 4% of patients having clinical diagnosis of IBS have CeD and an even larger percentage had potential CeD; thus, patients with IBS should be screened for CeD.

## 4. Non-Celiac Gluten Sensitivity (NCGS)

In the present decade, a new disease has resurfaced where a patient experiences both GI and non-GI symptoms with ingestion of gluten without any evidence of having either CeD or wheat allergy. This syndrome has been known as gluten sensitivity, gluten hypersensitivity, non-celiac gluten intolerance, however, currently the preferred name of this entity is non-celiac gluten sensitivity [18,41,42,43,44,45]. NCGS is characterized by intestinal symptoms (such as diarrhea, abdominal discomfort or pain, bloating, and flatulence) or extra-intestinal symptoms (such as headache, lethargy, attention-deficit/hyperactivity disorder, skin manifestations, or recurrent oral ulceration) [18,41].

While the number of publications on NCGS is increasing, the available literature on NCGS suffers from significant methodological flaws, mainly because of lack of validated diagnostic criteria for NCGS and use of non-validated outcome measures. Efforts have been made during the past five years to define the diagnostic criteria for NCGS as demonstrated by the Consensus Conferences on NCGS held in London (2011), Munich (2012) and Salerno (2015) and by several scientific contributions on this topic [18,41,42].

Nevertheless, considerable debate about NCGS has recently surfaced on the Internet, with a sharp increase in forums, patients and patient groups, manufacturers, and physicians advocating a gluten-free diet [41,42,43,44,45]. At present, the ratio between Google citations to PubMed citations for NCGS is 500:1, suggesting an increase of interest in NCGS by the general public. This clamor has now moved from the Internet to the popular press, where gluten has been named “the new diet villain”. Furthermore, a gluten-free diet has become the latest diet craze and is advocated and followed by many celebrities. There are general estimates that 10%–20% of people in USA and Australia are consuming gluten-free foods [41,42,43,44,45,46]. On the other hand, there is another school of thought that suggests that NCGS is largely imaginary and has been overestimated by patients and gluten-free food industry.

In summary, while there is a general agreement that NCGS does exist and its symptoms improve with the gluten-free diet for some patients. However, the detection of NCGS in these cases remains highly presumptive due to lack of a reliable diagnostic test.

## 5. Improvement in Symptoms of IBS with Gluten-Free Diet

Although NCGS has been recognized widely recently, Ellis and Linaker [47] first introduced the concept of NCGS almost 36 years ago, back in 1978, through a case-report published in the Lancet. They described a clinical story of a 43-year-old woman who had diarrhea, recurrent abdominal pain and abdominal distension for four months. Her investigations including examination of stool for ova, cysts, parasites, and occult blood, imaging investigations, sigmoidoscopic examination and jejunal mucosal biopsies were reported to be within normal limits. There was no definite response in her symptoms even with antibiotics and tranquillizers and her symptoms continued for two years. However, when a gluten-free diet was tried, her diarrhea stopped within four days and she showed improvement in other symptoms too. Resumption of the gluten-containing diet was followed by a rapid appearance of all her symptoms again. Such an experience was further validated by a dramatic relief in abdominal pain and chronic diarrhea in eight women once they were put on gluten-free diet and reappearance of symptoms upon their resumption of a gluten-containing diet [48]. After this remarkable demonstration of response in symptoms with the gluten-free diet in patients having diarrhea and abdominal pain (but not CeD), this entity remained obscure till the beginning of this decade.

There are reports suggesting that various oligo- and disaccharides, monosaccharides and polyols (FODMAPs, Fermentable oligo-disacchararides monosaccharides and polyols), rather than gluten, induce the abdominal symptoms in patients with NCGS, as a consequence of their resistance to digestion in the proximal small bowel, but susceptibility to digestion by bacteria in the distal small bowel and colon, suggesting that NCGS might not be a separate entity from IBS but rather a subgroup of IBS [18,30,46]. With the differences between the definition of food intolerance (GI symptoms secondary to fermentation of sugars by the colonic microbiota) and food hypersensitivity (an immune response to nutrient-derived antigens that causes GI and extra-GI symptoms), IBS and NCGS are distinct entities with some overlapping features. Moreover, although FODMAPs can cause GI symptoms such as bloating, they inhibit, rather than cause, intestinal inflammation [46]. Furthermore FODMAPs induce beneficial alterations in intestinal microbiota and generate short-chain fatty acids.

## 6. Does NCGS Exist in Patients with IBS?

Recently, Vazquez-Roque, *et al.* [49] demonstrated that gastrointestinal symptoms can be elicited by gluten in patients with diarrhea-predominant IBS (IBS-D), in whom CeD had been ruled out. They reported that patients with IBS-D had more bowel movements per day while they were on a gluten-containing diet than those maintaining a gluten-free diet; this aggravation of symptoms was most notable for those having HLA-DQ2 and/or -DQ8 haplotypes. Patients on a gluten-containing diet also had higher small intestinal permeability and under-expression of zonula occludens-1 in the small intestinal mucosa. There was no difference in the gastrointestinal transit time or mucosal histology for patients with IBS who were on the gluten-containing diet or on the gluten-free diet [49].

In another study from Australia, Biesiekierski, *et al.* [50] carried out a double-blind, placebo-controlled, re-challenge trial in which 34 patients with IBS-D were randomized. These patients, who had earlier demonstrated symptomatic relief with a gluten-free diet for at least 6 weeks before study enrollment, either received 16g of gluten per day via bread and a muffin (19 patients), or gluten-free bread and a gluten-free muffin (15 patients). Patients with CeD or other confounders were excluded from the study. The endpoint of the study was adequate symptom relief according to a questionnaire and visual analogue scale. After completion of the study, the investigators found that a significantly greater number of patients in the gluten-containing group did not experience adequate symptom control compared with the gluten-free group (68% *vs.* 40%; *p* = 0.001). Patients who received gluten-free diet reported significantly greater improvements in pain (*p* = 0.016), bloating (*p* = 0.031), satisfaction with stool consistency (*p* = 0.024), and tiredness (*p* = 0.001) in comparison to patients who ingested a diet that contained gluten.

In an interesting study, Fritcher-Ravens, *et al.* [51] using confocal laser endomicroscopy showed immediate epithelial breaks and leakage in response to a mucosal wheat challenge through the endoscope in 13 of 22 patients with food-related IBS in whom CeD and common food allergies were excluded. All positively identified patients later reported a dramatic, long-term (12 months) improvement on gluten-free diet.

In summary, current evidence suggests that symptoms of IBS improve with the gluten-free diet in about half of the patients tested.

## 7. What causes symptoms of IBS and NCGS?

A number of factors play a role in the pathogenesis of IBS, including: alterations in the brain-gut axis, genetic factors, impaired gut barrier function, immunologic dysregulation, changes in the gut microbiome, and psychosocial factors [1,52]. Patients with IBS frequently report exacerbation or triggering of symptoms by the ingestion of specific foods, and recently, there has been increasing attention on the role of dietary factors in the pathogenesis of IBS. It has been postulated that abnormal immune responses to dietary components might trigger symptoms in IBS [53,54,55,56]. In fact, more than 60% of patients with IBS report the onset or worsening of symptoms after meals (within 15 minutes of eating in 28% of patients and within three hours in 93% of patients) [53,54,55,56]. These are hypersensitivity reactions to food antigens, which can be either IgE mediated or non-IgE mediated. Patients with IBS having diarrhea appear to have increased colonic motility, particularly as indicated by and increase in the number of high amplitude propagating contractions (HAPCs) and in accelerated colonic transit, while those with constipation have reduced motility, fewer HAPCs, and delayed transit [52,57,58,59,60,61].

The alterations in the colonic motility are further supported by recent observations that postprandial platelet-depleted plasma 5-hydroxytryptamine concentration, a possible mediator of colonic motility, is increased in patients with diarrhea-predominant IBS, but reduced in those with constipation-predominant IBS [52,62].

While CeD predominantly involves the intestinal mucosa, abnormal systemic parasympathetic and sympathetic functions as well as intestinal dysmotility have been reported in CeD [63,64]. Although there are no direct evidences to suggest that patients with NCGS have intestinal dysmotility, certain evidences (as described below) may explain IBS type symptoms in these patients. An increase in acetylcholine release from the myenteric plexus and muscle hyper-contractility in response to gluten has recently been demonstrated in gliadin-sensitized HLA-DQ8 mice, which normalized after a gluten withdrawal. Furthermore, these motor changes in the animal model were accompanied by low-grade inflammation in the small intestine. The study suggests a mechanism by which gluten could induce gut motor dysfunction in the context of gluten sensitivity [65].

While the adaptive immune response to gluten peptide(s) is a major factor in the pathophysiological basis for CeD, many experts believe that the innate immune response to some wheat protein/peptides also plays a major role in the pathogenesis of NCGS [32,66,67,68]. Interestingly, a pepsin-trypsin gliadin digest has been shown to strongly activate myeloid cells *in vitro* (dendritic cells > macrophages > monocytes). The component of wheat flour that induced such activity was identified as the family of amylase-trypsin inhibitors (ATIs) [69]. ATIs engage toll-like receptor 4 (TLR-4) and release of pro-inflammatory cytokines in myeloid cells of both patients with CeD and non-diseased controls, as is expected for innate immune triggers. Mice deficient in TLR4 or TLR4 signaling are protected from the intestinal and systemic immune responses upon oral challenge with ATIs [32,69]. ATIs have been shown to increase intestinal and systemic release of cytokines and chemokines such as IL-8, TNF-α, and CCL-2 within 2–12 h in *in vivo* feeding experiments in mice [32,69].

ATIs are present in many plants, where they inhibit enzymes of common parasites such as mealworms and bugs in the wheat. ATIs also regulate metabolic processes occurring during seed development [70]. ATIs represent 2%–4% of total wheat protein (as compared to 80%–90% for gluten) [70].

An adult person usually consumes between 150 and 250 g of wheat flour per day and thus is exposed to 0.5–1 g of ATIs. Importantly, ATIs are present and even enriched in commercial gluten. ATIs resist proteolytic digestion by the gastric/ enteric proteases pepsin and trypsin, and are able to maintain their biological activity [32]. Plants other than wheat, rye, barley, and their early ancestors also contain inhibitors of amylase and trypsin-like activities, but show only minimal or no TLR4-activity [32]. The ATIs are not classified as gluten proteins, but ATIs are found in grain endosperm along with the gluten proteins and usually co-fractionate with the gliadin fraction, which complicates the situation. The designation of NCGS might be more appropriately be termed NCWS, non-celiac wheat sensitivity if the non-gluten proteins of wheat grain are proved to play a role in the sensitivity. A gluten-free diet is also supposed to be ATI free because avoidance of gluten necessarily involves avoidance of the implicated ATIs.

In summary, there is now evidence, although preliminary, that ATIs trigger innate immunity in CeD and NCGS.

## 8. Which Patients with IBS Should be Treated with Gluten-Free Diet?

At present, there are no defined predictors of response to a gluten-free diet in patients with IBS. Furthermore, there is no diagnostic marker for NCGS and the diagnosis is confirmed once symptoms improve with gluten exclusion and symptoms reappear with inclusion of gluten in the diet. A higher number of patients with NCGS have been shown to have HLA-DQ2/-DQ8 haplotype than the general population and response to gluten-free diet has been shown to be better in those having HLA-DQ2/DQ8 haplotype. Patients with IBS like symptoms should be screened for CeD using celiac-specific serological tests, such as anti-tissue transglutaminase antibody, anti-endomysial antibody or anti-deamidated gluten peptide and those having a positive test should undergo duodenal mucosal biopsies. If this testing indicates the presence of villous abnormalities corresponding to a modified Marsh grade 2, or more, this confirms the presence of CeD and the patient should be put on gluten-free diet. If the celiac-specific serological tests are negative in symptomatic patients, a positive anti-gliadin antibody may be an indicator of NCGS and a trial of a gluten-free/wheat-free diet may be administered to these patients. An improvement in symptoms while on a gluten free/wheat free diet would tend to support the diagnosis of NCGS.

## 9. Unifying Hypothesis and Conclusions

As discussed above, both IBS and NCGS are common in the general population and therefore both can coexist with each other independently without necessarily sharing a common pathophysiological basis. While the treatment of NCGS is exclusion of gluten from the diet, some of the patients with IBS do improve with the gluten-free diet. Furthermore, minimal inflammation in the gut has been demonstrated in both IBS and NCGS. It is thus conceivable that ingestion of wheat containing ATIs, in the presence of intestinal inflammation enhances an innate immune response that plays a role in the generation of symptoms in patients with IBS, which then resolve when the patient takes up a gluten-free/wheat free diet. Furthermore, the microbiome may also play a role in the pathogenesis of NCGS.
